# Prevalence of Asthma and Asthma-like Symptoms: a Study in Five Provinces of Iran

**Published:** 2019-04

**Authors:** Hooman Sharifi, Mostafa Ghanei, Hamidreza Jamaati, Mohammad Reza Masjedi, Hadis Najafimehr, Atefeh Fakharian, Alireza Eslaminejad

**Affiliations:** 1 Tobacco Prevention and Control Research Center, National Research Institute of Tuberculosis and Lung Diseases (NRITLD), Shahid Beheshti University of Medical Sciences, Tehran, Iran; 2 Chemical Injuries Research Center, Systems Biology and Poisonings Institute, Baqiyatallah University of Medical Sciences, Tehran, Iran; 3 Chronic Respiratory Diseases Research Center, NRITLD, Shahid Beheshti University of Medical Sciences, Tehran, Iran; 4 Tobacco Control Research Center, Iranian Anti-Tobacco Association, Tehran, Iran; 5 Gastroenterology and Liver Diseases Research Center, Research Institute for Gastroenterology and Liver Diseases, Shahid Beheshti University of Medical Sciences, Tehran, Iran.

**Keywords:** Asthma, Asthma-like Symptoms, Iran

## Abstract

**Background:**

Asthma is a complex chronic inflammatory airway disease affecting millions of people worldwide. The prevalence of asthma attacks in most regions of the world, including the developing countries, increases due to urbanization, industrialization, and lifestyle.

**Materials and Methods:**

The present study aimed at investigating the prevalence of asthma and asthma symptoms in five provinces of Iran using the stratified cluster sampling method and the European Community Respiratory Health Survey (ECRHS) questionnaire.

**Results:**

A total of 4918 subjects were enrolled in the study. The prevalence of nocturnal cough was 27.4% (95% confidence interval (CI): 26%–28%); it was the most common asthma symptom followed by nocturnal shortness of breath (19.6%; 95%CI: 18–21%). For participants aged 20–44 years, the most prevalent symptoms were coughing attacks (37.7%), shortness of breath (26.5%), and nasal allergies (22.7%), respectively.

**Conclusion:**

There was a significant association among gender, age, and nasal allergy. Relevant studies should be conducted to figure out the countrywide distribution and the real burden of the disease.

## INTRODUCTION

Asthma is a complex chronic inflammatory airway disease affecting millions of people worldwide. The overall prevalence of asthma is difficult to estimate precisely, ranging from 1% to 20% using different case definitions for both children and adults (1, 2). According to the Global Asthma Report, the prevalence of asthma attacks and treatment for asthma increase in most regions of the world including the developing countries due to urbanization, industrialization, lifestyle, increased awareness of wheeze and wheeze-related symptoms, and also in response to international guidelines for asthma treatment during the 1990s (3, 4). Evaluation of asthma usually involves a combination of signs and symptoms, including patients’ medical history, physical examination, and lung function test. Clinical definitions given in questionnaires are usually used in prevalence studies (5–8).

In Iran, the prevalence of asthma in children and adolescents was investigated during the last decade. Shokouhi et al., in a cross sectional study on 2569 subjects reported the prevalence of asthma as 7.6% (9). Tazesh et al., in a survey in Tehran, revealed that 10.8% of inhabitants aged 20–44 years had asthma (wheezing and breathlessness) (10). In two other studies conducted in Mashhad and Urmia, the prevalence of asthma symptoms was less than the reported figures (11, 12). In the authors’ previous study in Tehran (2016), using the European Community Respiratory Health Survey (ECRHS) questionnaire, 6.7% of participants had wheezing and breathlessness (13) A meta-analysis by Masjedi et al., evaluated articles published on asthma using data from 23 countries of EMRO. They reported a 7.95% prevalence of asthma in Iran that was lower than those of Kuwait, Qatar, and Saudi Arabia in the region (14).

The ECRHS is wildly used in different regions to address asthma epidemiology in adults (6, 15). The ECRHS was the first study to assess the status of asthma in adults in 25 countries in three phases in terms of geographical prevalence, risk factors, treatment, and follow-up (5, 16, 17). The questionnaire is now one of the most popular instruments for epidemiologic studies due to its high validity and acceptability. It contains 10 general and specific questions used in large-scale surveys of asthma. It is advantageous to study asthma distribution and prevalence using the same instruments and definitions, including comparison with other studies, accurate estimation of the burden of the disease, and formulation of appropriate prevention policies.

The present study aimed at investigating the prevalence of asthma using the ECRHS questionnaire and measuring the prevalence of asthma symptoms among adults aged 20 to 44 years in five provinces of Iran.

## MATERIALS AND METHODS

### Population and sampling strategy

The current population-based, cross sectional study was conducted to assess the frequency of asthma and asthma symptoms in five provinces of Iran with 28.7 million populations, nearly one-third of the Iran population. In the current study, the country was divided into five geographical areas: North, East, West, South, and Center. The stratified cluster sampling method was used considering urban areas and the density of the population in each city.

Sample Size: According to the authors’ previous study, (13) considering the study power of at least 80%, the effect size of 1.5, and the response rate of 60%, the sample size was calculated 961. The sample size was determined 3366 to obtain accurate results. Finally, 4918 subjects were enrolled in the study.

Sampling: As mentioned above, (18) the stratified cluster sampling method was utilized for proportional allocation using Strata. The target population included all male and female Iranian individuals aged 20 to 44 years living in Tehran, Mazandaran, Khorasan Razavi, Kerman, and Khuzestan provinces. The exclusion criterion was the unwillingness to participate in the study. The participants were recruited via two ways in terms of age allocation. First, the participants were assigned to three age groups, including <20, 20–39, and >40 years in order to evaluate demographic characteristics, frequency of relevant symptoms, and medical history. Then, the participants were studied if they were in the age range of 20 to 44 years, considering mandatory standards in ECRHS screening. In order to minimize the misclassification regarding chronic obstructive pulmonary diseases (COPD), spirometry data were collected, which expanded the population age range beyond ECRHS to some extent.

The stratification was performed considering municipal districts of the administrative center of provinces. Appreciating the population density in different districts, the appropriate number of clusters was weighted for each district. The number was also affected by the total sample size, the average number of members per household, logistics facilities for subject enumeration, transport, and examination. There were three-member teams to interview with the clusters in order to obtain data. Two interviewers, a man and a woman dressed in white medical coats, and a driver were recruited. The interviewing team approached the index household specified via the random selection of clusters and continued the enumeration in 10 neighboring households in a systematic manner by proceeding in a clockwise direction. The interviewers were advised to try the Kish grid to select the right participant(s) when there was more than one member in the indexed household. The Kish grid is a table of numbers used to find the number of residents in the household. Then, a randomly selected number determines the interviewee.

Definition: Two definitions used in previous studies on asthma prevalence were retrieved. Asthma symptoms in the study were considered based on the following definitions:

1. ECRHS (15, 19), which implies possible asthma: Woken up by breathlessness attacks, history of an asthma attack, or currently taking asthma medications within the preceding 12 months.
2. Modified ECRHS: (20) Presence of the symptoms wheezing or whistling, shortness of breath attack, diagnosed asthma attack in the past 12 months, or currently taking medicines for asthma.

Current asthma was defined as affirmative answers to each of the following questions: “Have you ever had asthma?” followed by “Was this confirmed by a doctor?”, and “Have you had at least one asthma symptom in the past 12 months?” (5). If a respondent was diagnosed with asthma by a physician and experienced asthma symptoms in the past 12 months, he/she was defined as currently having asthma.

Examination protocol and questionnaire: The main instrument to estimate the prevalence of asthma and asthma symptoms was the ECRHS questionnaire (16) previously used in similar domestic studies (11). The ECRHS questionnaire is the standard tool used in the survey of the European Respiratory Society at 48 centers in 17 European countries and five non-European countries from 1990 to 1995 in adults aged 20–44 (16). Back-translation from Farsi into English version was performed to confirm the questionnaire’s validity.

All questionnaires were filled out by interviewers. To evaluate asthma symptoms, the same questions included in the ECRHS were asked.

### Statistical analysis

Frequency tables, including number and percentage, were used for qualitative variables and mean and standard deviation (SD) were also employed for continuous variables. The prevalence was reported as a proportion, with a 95% confidence interval (CI). For intergroup comparisons, analysis of variance (ANOVA) was used, and to compare continuous and qualitative variables; the Chi-squared test was utilized. Statistical analyses were performed by SPSS version 19; P-value <0.05 was considered as the level of significance.

The materials and methods of the study were published in detail elsewhere. (18)

## RESULTS

A total of 4918 subjects were enrolled in the study (42.6% male and 57.4% female). The mean age of the subjects was 56.11±30.06 years. The number of participants who determined the smoking status was 977 of which 193 were smokers. Table 1 presents the participants’ characteristics by age.

**Table 1.** **Demographic** characteristics of participants by age

**Variable**	**<20 yrs. n=6**	**20–39 yrs. n=2279**	**40 yrs. or more n=1066**	**Unknown n=1567**	**Total**
Male gender, n(%)	2(33.3)	1263(55.5)	480(45.1)	24(3.0)	1769(42.6)
Mean age years ±SD	13.16±6.46	31.90±4.6	45.07±5.72	-	56.11±30.06
Smoking, n(%)	1(33.3)	129(19.5)	34(12.2)	29(54.7)	193(19.4)

### Prevalence of respiratory symptoms and use of asthma medications:

The prevalence of nocturnal cough was 27.4% (95%CI: 26%–28%); it was the most common asthma symptom followed by nocturnal shortness of breath 19.6% (95%CI: 18%–21%). Also, 2.3% (95%CI: 2–3 %) had received an active asthma diagnosis (an asthma attack in the past 12 months and the current use of asthma medication).

### Prevalence of asthma using different definitions:

The estimated prevalence was different depending on the definition. It was 20.6% (95% CI: 19.5–21.7 %) and 30.7% (95% CI: 29.5–31.9%) based on ECRHS and modified ECRHS, respectively.

Prevalence of asthma symptoms according to modified

### ECRHS:

The asthma symptoms are presented in Table 2. The most prevalent symptom was nasal allergies (29.5%), followed by a coughing attack (27.4%), and shortness of breath (19.6%). Also, the asthma symptoms were stratified by age; symptoms among the age groups were significantly different (P <0.05).

**Table 2.** Symptoms according to the modified ECRHS screening questionnaire in participants, by age

**ECRHS screening questionnaire symptoms within the last 12 months, n (%)**	**<20 or unknown yr. n=1566**	**20–39 yr. n=2279**	**40 yr. or more n=1069**	**Total n=4912**	**P- value**
Wheezing/whistling	230(14.8)	448(19.7)	248(23.2)	926(18.9)	<0.001
Wheezing with breathlessness	228(14.6)	134(5.9)	101(9.5)	463(9.4)	<0.001
Wheezing without a cold	403(25.7)	294(12.9)	161(15.1)	858(17.5)	<0.001
Chest tightness	81(5.2)	488(21.5)	248(23.4)	817(16.7)	<0.001
Have you ever experienced shortness of breath?	61(3.9)	585(25.9)	311(29.3)	957(19.6)	<0.001
Attack of coughing	104(6.6)	854(37.6)	383(36.0)	1341(27.4)	<0.001
Are you informed by the physician that you got asthma?	2(0.1)	68(3.0)	41(3.9)	111(2.3)	<0.001
Currently taking asthma medications	2(0.1)	50(2.2)	28(2.6)	80(1.6)	<0.001
Nasal allergies (including hay fever)	709(45.5)	515(22.7)	216(20.3)	1440(29.5)	<0.001

### Prevalence of asthma symptoms for patients with wheezing, coughing, asthma, or nasal allergies:

Totally 2169 participants responded “YES” to Q1 wheezing, Q4 coughing, Q5 asthma, or Q7 nasal allergies. In these subjects, females were older than males with the mean age of 57.86 and 35.75 years, respectively (P <0.001). The number of participants in the 20–39 years age group was greater than those of the other age groups (P <0.001). The prevalence of asthma symptoms by gender for the ones who answered YES is shown in Table 3. Coughing attack (58.9%), nasal allergies (47.5%), and wheezing (39.1%) were the most prevalent symptoms, respectively.

**Table 3.** Characteristics of those responding “YES” to Q1 “wheezing”, Q4 “coughing”, Q5 “asthma”, or Q7 “nasal allergies” according to the ECRHS screening questionnaire (n =2169)

**Variable**	**Male (n=926)**	**Female (n=1243)**	**Total (n=2169)**	**P-value**
Age in years, mean (SD)	35.75(10.89)	57.86(29.96)	46.80(20.5)	<0.001
Age Groups				<0.001
Unknown or <20	14(1.5)	419(33.7)	433(20.0)	
20–39	671(72)	520(41.9)	1191(54.9)	
40 or more	241(26.0)	303(24.4)	544(31.5)	
Wheezing/whistling	410(44.3)	437(35.2)	847(39.1)	<0.001
Have you ever experienced shortness of breath?	311(33.9)	389(31.5)	700(32.5)	0.24
Attack of coughing	663(71.6)	614(49.4)	1277(58.9)	<0.001
Are you informed by the physician that you got asthma?	56(6.0)	53(4.3)	109(5.0)	0.07
Currently taking asthma medications	38(4.1)	36(2.9)	74(3.4)	0.12
Nasal allergies (including hay fever)	367(39.6)	664(53.4)	1031(47.5)	<0.001

### Prevalence of modified ECRHS in participants aged 20–44 years:

For participants aged 20–44 years, the most prevalent symptoms were coughing attack (37.7%), shortness of breath (26.5%), and nasal allergies (22.7%), respectively. The coughing attack was also the most prevalent symptom in stratifying by gender. The prevalence of males and females was 38.9% and 36.2%, respectively. The prevalence of wheezing and shortness of breath was significantly different between males and females (P <0.05) (Table 4).

**Table 4.** Symptoms according to the modified ECRHS screening questionnaire in participants 20–44 yr, by gender

**ECRHS screening questionnaire symptoms within the last 12 months, n (%)**	**20–44 yr. Male n=1556**	**20–44 yr. Female n=1336**	**Total n=2898**	**P-value**
Wheezing/whistling	359(23.1)	239(17.9)	598(20.7)	0.001
Wheezing with breathlessness	116(7.5)	71(5.3)	187(6.5)	0.02
Wheezing without a cold	244(15.7)	143(10.7)	387(13.4)	<0.001
Chest tightness	368(23.7)	274(20.6)	642(22.3)	0.05
Have you ever experienced shortness of breath?	361(23.3)	402(30.3)	763(26.5)	<0.001
Attack of coughing	603(38.9)	483(36.2)	1086(37.7)	0.15
Are you informed by the physician that you got asthma?	48(3.1)	42(3.2)	90(3.1)	1.00
Currently taking asthma medications	35(2.3)	29(2.2)	64(2.2)	0.90
Nasal allergies (including hay fever)	337(21.7)	318(23.9)	655(22.7)	0.18

### The trend in the prevalence of wheezing and breathlessness in males and females according to age:

Figures 1 and 2 indicate the trend in wheezing and breathlessness by age and gender. The prevalence of wheezing for males increased up to 30% and then decreased for older participants; for females, wheezing increased up to 40% and then showed a decreasing trend. The prevalence of shortness of breath was higher among females than males, based on age.

### Factors associated with asthma symptoms according to ECRHS and modified ECRHS screening:

According to ECRHS screening, gender, and a history of nasal allergy had statistically significant relationships with asthma symptoms. The chance of asthma for females was higher than that of males (odds ratio (OR): 1.34; 95%CI: 1.15–1.57). Also, the chance of asthma for subjects with nasal allergy was 69% more than that of the ones without nasal allergy (OR: 1.69; 95%CI: 1.42–2.02).

For modified ECRHS, the history of nasal allergy was the only factor associated with asthma symptoms (OR: 2.65; 95%CI: 1.92–3.66), and the chance of asthma was twice more for patients with allergy than the ones with no allergic reactions (Table 5).

**Table 5.** Predictors of asthma using the different asthma definitions

**Predictors**	**ECRHS**		**Modified ECRHS**	

	**OR(95%CI)**		**OR(95%CI)**	
	**Univariate**	**Multivariate**	**Univariate**	**Multivariate**
Age				
20–39	1		1	
>=40	1.13(0.96,1.33)		1.11(0.95,1.29)	
Sex (female)	1.37(1.17,1.60)	1.34(1.15,1.57)	1.06(0.92,1.22)	
History of Nasal allergy	1.71(1.43,2.04)	1.69(1.42,2.02)	2.33(1.97,2.76)	2.65(1.92,3.66)
Cigarette smoking	1.49(0.87,2.55)		1.51(1.05,2.17)	1.41(0.97,2.05)

## DISCUSSION

In the current study, the prevalence of asthma and asthma symptoms were addressed in Iranian adults living in Tehran, Mazandaran, Khorasan Razavi, Kerman, and Khuzestan provinces. The study mainly found that the estimated prevalence of asthma symptoms varied from 20.6% to 30.7%, depending on definition. The asthma definition used in the current study considered three risk factors, while the other definitions considered four. It seems that the main cause of variations was the sensitivity of the definitions as a diagnostic tool. The currently available asthma definitions have their own pros and cons, and there is no gold standard definition for asthma. (21, 22) However, to the best of authors’ knowledge, the lack of a standard operational definition for asthma in adults may lead to a potential problem in international comparisons, the accurate burden of disease, and healthcare resource planning.

According to the obtained results, 2.2% of participants aged 20 to 44 years used asthma medications. It was 4.7% and 3% in the studies by Fazlollahi (23) et al., and Shokouhi Shoormasti (9) et al., respectively. The prevalence of taking asthma medications was 2.6% in the study by Baççıoğlu et al., in Turkey (24). Other studies reported various prevalence for taking asthma medications from 1.3% to 5.1%, which was in agreement with the current study findings (25, 26). The prevalence of taking asthma medications did not vary by gender in the current study, which could be due to the relatively narrow age group studied. This finding suggested that other factors, such as physician’s practice can affect the selected treatment.

There are several reports on the prevalence of asthma symptoms in different countries. The prevalence of wheezing was 8.6% in 70 countries, ranging from 1.73% in China to 27.4% in Australia (27). According to the reviewed literature, the prevalence of wheezing in Turkey, Pakistan, and the UAE was 11.34%, 5.02%, and 7.21%, respectively. The current study findings showed a higher prevalence of wheezing (20.7%) in participants aged 20 to 44 years compared with those of some Asian countries and a lower prevalence than European countries. Although some studies conducted in Iran reported no significant differences in the prevalence of wheezing between genders (9, 23), the present study findings suggested that the female gender was more prone to develop wheezing, similar to the results of Johannessen et al. (28), in a long-term follow-up in ECRHS. The same results were reported in the study by Stern et al., in the US, indicating that newly diagnosed asthma was twice more likely to occur in females than males (29). However, based on such findings, the assumption can be made that the incidence of asthma partly depends on gender.

Moreover, the current study observed a decade difference between the pick of wheezing in both genders. This finding could suggest a gradual change in the prevalence of wheezing between males and females in early adulthood and remission of the situation in the third decade of life for males and the fourth decade for females. Though the proposition cannot be extended to current asthma, wheezing could manifest itself as the crucial symptom of asthma in early adulthood. These findings verified and extended the reports of some studies suggesting a gradual change in the relative prevalence of asthma in males and females between the pubertal years and early adulthood (30–32).

The present study evaluated the risk of asthma symptoms associated with age, gender, history of nasal allergy, and cigarette smoking in a population-based setting. The chance of asthma symptoms significantly differed between males and females, which might be due to a different number of male and female subjects included in the study. As discussed earlier, the reasons underlying the distinctive gender differences need further investigation. Also, the prevalence of asthma among subjects with nasal allergy was 69% higher than that of the ones without it. This finding was in agreement with those of the other studies that observed a clear association between nasal allergy and asthma or asthma symptoms (20, 33). Desalu et al. revealed that nasal allergy and a family history of nasal allergy were the most common symptoms and predictors of asthma (34).

The main strengths of the study were the large and representative sample size and the use of a standardized and validated screening questionnaire. In addition, a rigorous sampling method was used, and the response rate was high.

## CONCLUSION

In conclusion, according to the findings of the current study, the prevalence of asthma symptoms was considerably high in the five largest provinces of Iran in comparison with those of other countries. Moreover, there was a significant association among gender, age, and nasal allergy. Relevant studies should be conducted to understand the countrywide distribution and the real burden of the disease.

## References

[1] Masoli M Fabian D Holt S Beasley R Global Initiative for Asthma (GINA) Program The global burden of asthma: executive summary of the GINA Dissemination Committee report. Allergy 2004;59(5):469–78. 15080825 10.1111/j.1398-9995.2004.00526.x

[2] Desalu OO Oluboyo PO Salami AK The prevalence of bronchial asthma among adults in Ilorin, Nigeria. Afr J Med Med Sci 2009;38(2):149–54. 20175418

[3] The Global Asthma Report 2014. Auckland, New Zealand: Global Asthma Network; 2014. [June 19, 2016]. Available at http://www.globalasthmareport.org/reseources/Global_Asthma_Report_2014.pdf.

[4] Chinn S Jarvis D Burney P Luczynska C Ackermann-Liebrich U Antó JM Increase in diagnosed asthma but not in symptoms in the European Community Respiratory Health Survey. Thorax 2004;59(8):646–51. 15282382 10.1136/thx.2004.021642 PMC1747094

[5] Janson C Chinn S Jarvis D Burney P Physician-diagnosed asthma and drug utilization in the European Community Respiratory Health Survey. Eur Respir J 1997;10(8):1795–802. 9272921 10.1183/09031936.97.10081795

[6] Janson C Anto J Burney P Chinn S de Marco R Heinrich J The European Community Respiratory Health Survey: what are the main results so far? European Community Respiratory Health Survey II. Eur Respir J 2001;18(3):598–611. 11589359 10.1183/09031936.01.00205801

[7] Asher MI Keil U Anderson HR Beasley R Crane J Martinez F International Study of Asthma and Allergies in Childhood (ISAAC): rationale and methods. Eur Respir J 1995;8(3):483–91. 7789502 10.1183/09031936.95.08030483

[8] Akinbami LJ Moorman JE Bailey Zahran HS King M Johnson CA Trends in asthma prevalence, health care use, and mortality in the United States, 2001–2010. NCHS data brief. 2012; 94: pp. 1–8. 22617340

[9] Shokouhi Shoormasti R Pourpak Z Fazlollahi MR Kazemnejad A Nadali F Ebadi Z The Prevalence of Allergic Rhinitis, Allergic Conjunctivitis, Atopic Dermatitis and Asthma among Adults of Tehran. Iran J Public Health 2018;47(11):1749–1755. 30581793 PMC6294865

[10] Tazesh B Shaabani A Fazlollahi MR Entezari A Dashti R Pourpak Z Prevalence of asthma symptoms and smoking behavior among 20–44 years old adults in Tehran: A telephone survey. Health 5(3): 469–474.

[11] Rahimi-Rad MH Gaderi-Pakdel F Salari-Lak S Smoking and asthma in 20–44-year-old adults in Urmia, Islamic Republic of Iran. East Mediterr Health J 2008;14(1):6–16. 18557447

[12] Varasteh AR Fereidouni M Shakeri MT Vahedi F Abolhasani A Afsharian MS Samei M Sankian M Prevalence of allergic disorders among the population in the city of Mashhad, Northeast Iran. Journal of Public Health 2009;17(2):107–12.

[13] Sharifi H Ghanei M Sadr M Emami H Fakharian A Hessami Z Prevalence and Geographic Distribution Pattern of Asthma in Tehran by ECRHS. Tanaffos 2016;15(4):236–242. 28469680 PMC5410120

[14] Masjedi M Ainy E Zayeri F Paydar R Assessing the Prevalence and Incidence of Asthma and Chronic Obstructive Pulmonary Disease in the Eastern Mediterranean Region. Turk Thorac J 2018;19(2):56–60. 29755807 10.5152/TurkThoracJ.2018.17051 PMC5937810

[15] Janson C Chinn S Jarvis D Burney P Physician-diagnosed asthma and drug utilization in the European Community Respiratory Health Survey. Eur Respir J 1997;10(8):1795–802. 9272921 10.1183/09031936.97.10081795

[16] Burney PG Luczynska C Chinn S Jarvis D The European Community Respiratory Health Survey. Eur Respir J 1994;7(5):954–60. 8050554 10.1183/09031936.94.07050954

[17] Variations in the prevalence of respiratory symptoms, self-reported asthma attacks, and use of asthma medication in the European Community Respiratory Health Survey (ECRHS). Eur Respir J 1996;9(4):687–95. 8726932 10.1183/09031936.96.09040687

[18] Sharifi H Masjedi MR Emami H Ghanei M Buist S Burden of obstructive lung disease study in tehran: research design and lung spirometry protocol. Int J Prev Med 2014;5(11):1439–45. 25538840 PMC4274551

[19] Obaseki DO Awoniyi FO Awopeju OF Erhabor GE Low prevalence of asthma in sub Saharan Africa: a cross sectional community survey in a suburban Nigerian town. Respir Med 2014;108(11):1581–8. 25443397 10.1016/j.rmed.2014.09.022

[20] Erhabor GE Obaseki DO Awopeju OF Ijadunola KT Adewole OO Asthma in a university campus: a survey of students and staff of Obafemi Awolowo University, Ile-Ife, Nigeria. J Asthma 2016;53(1):30–6. 26313508 10.3109/02770903.2015.1060609

[21] European Community Respiratory Health Survey: Symptom outcome measures in the ECRHS. http://www.ecrhs.org/Publications/symptomoutcomes.pdf.

[22] Pekkanen J Sunyer J Anto JM Burney P European Community Respiratory Health Study Operational definitions of asthma in studies on its aetiology. Eur Respir J 2005;26(1):28–35. 15994386 10.1183/09031936.05.00120104

[23] Fazlollahi MR Najmi M Fallahnezhad M Sabetkish N Kazemnejad A Bidad K The prevalence of asthma in Iranian adults: The first national survey and the most recent updates. Clin Respir J 2018;12(5):1872–1881. 29227026 10.1111/crj.12750

[24] Baççıoğlu A Söğüt A Kılıç Ö Beyhun E The Prevalence of Allergic Diseases and Associated Risk Factors in School-Age Children and Adults in Erzurum, Turkey. Turk Thorac J 2015;16(2):68–72. 29404081 10.5152/ttd.2015.4229 PMC5783063

[25] Tug T Acik Y Prevalence of asthma, asthma-like and allergic symptoms in the urban and rural adult population in Eastern Turkey. Asian Pac J Allergy Immunol 2002;20(4):209–15. 12744620

[26] Demir AU Kalyoncu AF Selçuk T Artinli M Sahin A Prevalence of Asthma, Allergy and respiratory symptoms in Hasançelebi/Hekimhan/Malatyian Eastern Turky. Tur Respir J 2001;2(2):29–34.

[27] To T Stanojevic S Moores G Gershon AS Bateman ED Cruz AA Global asthma prevalence in adults: findings from the cross-sectional world health survey. BMC Public Health 2012;12:204. 22429515 10.1186/1471-2458-12-204 PMC3353191

[28] Johannessen A Verlato G Benediktsdottir B Forsberg B Franklin K Gislason T Longterm follow-up in European respiratory health studies - patterns and implications. BMC Pulm Med 2014;14:63. 24739530 10.1186/1471-2466-14-63 PMC4021078

[29] Stern DA Morgan WJ Halonen M Wright AL Martinez FD Wheezing and bronchial hyper-responsiveness in early childhood as predictors of newly diagnosed asthma in early adulthood: a longitudinal birth-cohort study. Lancet 2008;372(9643):1058–64. 18805334 10.1016/S0140-6736(08)61447-6 PMC2831297

[30] Nicolai T Pereszlenyiova-Bliznakova L Illi S Reinhardt D von Mutius E Longitudinal follow-up of the changing gender ratio in asthma from childhood to adulthood: role of delayed manifestation in girls. Pediatr Allergy Immunol 2003;14(4):280–3. 12911505 10.1034/j.1399-3038.2003.00047.x

[31] Ownby DR Johnson CC Peterson EL Incidence and prevalence of physician-diagnosed asthma in a suburban population of young adults. Ann Allergy Asthma Immunol 1996;77(4):304–8. 8885808 10.1016/S1081-1206(10)63325-X

[32] Wright AL Stern DA Kauffmann F Martinez FD Factors influencing gender differences in the diagnosis and treatment of asthma in childhood: the Tucson Children’s Respiratory Study. Pediatr Pulmonol 2006;41(4):318–25. 16477658 10.1002/ppul.20373

[33] Al Ghobain MO Al-Hajjaj MS Al Moamary MS Asthma prevalence among 16- to 18-year-old adolescents in Saudi Arabia using the ISAAC questionnaire. BMC Public Health 2012;12:239. 22443305 10.1186/1471-2458-12-239 PMC3384472

[34] Desalu OO Sanya EO Adeoti AO Aderibigbe SA Kolo PM Impact of Operational Definitions on the Predictors and Prevalence of Asthma Estimates: Experience from a University Students’ Survey and Implications for Interpretation of Disease Burden. Ethiop J Health Sci 2018;28(6):725–734. 30607089 10.4314/ejhs.v28i6.7 PMC6308751

